# Fabrication of an electrochemical sensor based on eggshell waste recycling for the voltammetric simultaneous detection of the antibiotics ofloxacin and ciprofloxacin

**DOI:** 10.1186/s13065-023-01044-8

**Published:** 2023-09-30

**Authors:** M. Khodari, H. F. Assaf, Ahmed A. Shamroukh, E. M. Rabie

**Affiliations:** Chemistry Department, Faculty of Science, South Valley University, Qena, 83521 Egypt

**Keywords:** Electroanalytical methods, Chemical sensors, COVID-19, Nanostructure, Ciprofloxacin, Ofloxacin

## Abstract

In this work, an accurate, highly sensitive, and economical electrochemical sensor based on a carbon paste electrode modified by Ca_2_CuO_3_ nanostructure (Ca_2_CuO_3_ NS) was constructed using Eggshell waste recycling as a cheap source of calcium. The Ca_2_CuO_3_ NS was analyzed using FTIR, SEM, and XRD measurements. The synthesized nanomaterials utilized for the first time to enhance the electrocatalytic efficiency of carbon paste electrode (CPE) toward fluoroquinolones antibiotics ofloxacin (OFL) and ciprofloxacin (CIP), The drugs used to treat pneumonia caused by COVID-19. The synthesized Ca_2_CuO_3_ NS dramatically enhanced the anodic peak response of CPE toward both drugs compared to the unmodified one and other modified electrodes. The simultaneous detection of the two antibiotics was performed in the linear range of 0.09–1.0 μM for OFL and 0.05–0.8 μM for CIP with the LOD of 0.027 μM and 0.012 μM, respectively. The suggested method was applied successfully to determine OFL and CIP in real samples.

## Introduction

Fluoroquinolones (FQs), one of the most important classes of synthetic antibiotics, exhibit a broad-spectrum antibacterial effect, including Gram-negative and Gram-positive microorganisms [1, 2].Various FQs with various structural variations exhibit the same therapeutic efficacy because they depend on inhibiting bacterial DNA gyrase [3]. Because of their great activity, several FQs are frequently employed in hospitals, animal husbandry, and aquatic farming to prevent different diseases. Recently, many instances involving the use of various FQs in managing and treating COVID-19 have been reported worldwide [4]. Second-generation FQs of ofloxacin and ciprofloxacin are frequently prescribed to treat gonorrhoea, peritonitis, respiratory tract infections, osteomyelitis, gastrointestinal and soft tissue infections, and infections of the skin and other body tissues due to their high potency, low minimal inhibitory concentration, low toxicity, the long half-life, and high stability [1–5].

Structures of FQs are very similar; thus, it can be challenging to detect them simultaneously. However, several techniques, including spectroscopy [6, 7], chromatographic methods [8–10], and capillary electrophoresis [11], have been reported to identify FQs. These techniques can identify and separate FQs, but their general use is constrained by their complicated sample pretreatment procedures and pricey equipment. On the other hand, the electrochemical analysis approach has lately been used for FQs measurement due to its simplicity, speedy assay time, and affordable instrumentation [12, 13].

Carbon paste electrode (CPE) was frequently utilized as a working electrode in electroanalytical techniques because of its reusable surface, low cost, and simplicity of modification with a variety of materials, which allows the enhancement of the sensitivity and selectivity by increasing its activated surface area [14, 15]. To improve the sensing performance of CPE and to speed up the electron transfer rate for various redox systems, several metal oxide nanoparticles have recently been used to modify the surface of the CPE [16].

Recently, several nanocomposites have been utilized as a modifier to improve the performance of sensing electrodes, due to their superior chemical and physical properties such as morphology, electroactivity, and conductivity [17–19]. Cu-containing oxide materials such as Ca_2_CuO_3_ and CaCu_2_O_3_ are significant types of multi-metal oxide materials that have received much attention owing to their unique features, including superconductivity and optical transparency. These systems stand out due to their more vital catalytic activity than pure components and larger surface area than pure oxides [20, 21].

Eggshells are a regular food waste that is generated in enormous quantities every day worldwide. The eggshell treatment is considered a cheap calcium source since CaCO_3_ makes up a significant percentage of eggshell waste (more than 97%) [22, 23]. The manufacture of Ca_2_CuO_3_ NS in this study utilized eggshell waste as a natural, affordable, and biodegradable source of Ca. The mixed oxide produced was used to improve CPE's ability to simultaneously detect OFL and CIP antibiotics in real samples.

Here, a novel, accurate, highly sensitive, and economical electrochemical sensor based on eggshell waste recycling for the voltammetric detection of the antibiotics ofloxacin and ciprofloxacin was fabricated. Our strategy is based on eggshell waste recycling to extract Ca_2_CuO_3_ nanostructure, which in turn was used, for the first time, to modify CPE. The simultaneous detection of OFL and CIP antibiotics was achieved. The constructed sensor was successfully functionalized under optimal conditions for simultaneous sensing OFL and CIP in human serum and commercial pharmaceutical tablets with an acceptable recovery value (97.32% to 100.40%).

## Experimental procedures

### Materials

Pure OFL (≥ 99%) and CIP (≥ 98%) were purchased from Sigma-Aldrich (UK). Cupric chloride (powder, 99%) purchased from Merck (Germany). Graphite fine powder (98%) was purchased from LOBA Chemie company (India). Other reagents and chemicals of analytical grade were used in this investigation without further purification. Deionized water was used to prepare the utilized aqueous solutions. A refrigerator was used to store various stock solutions until usage in the lab. Daily prepared phosphate buffer solution (PBS) was applied as a supporting electrolyte.

### Instruments

All electrochemical studies were carried out using Versa STAT4 and a three-electrode cell consisting of Ca_2_CuO_3_ NS/CPE, Ag/AgCl, and Pt wire as the working electrode, the reference electrode, and the counter electrode, respectively. A (Perkin Elmer) spectrometer was used for FT-IR analysis, and an XRD record was obtained at 25 °C employing (Brucker D8 Advance, Germany). SEM was used to examine the sample's surface morphology (JSM-5500 LV, Japan).

### Synthesis of Ca_2_CuO_3_ powder

The coprecipitation method was utilized to prepare Ca_2_CuO_3_ powder, as described previously [24], with some modifications. In brief, the eggshell powder was prepared according to our previous work in the first step [22]. Then by considering that about 97% of eggshell waste consists of CaCO_3_ [25], the appropriate weight of the resulting powder was dissolved in 100 ml HCl (1.0 M) to form CaCl_2_ (1.0 M) solution. It was added to 100 ml of CuCl_2_ (0.5 M), followed by the dropwise addition of NaOH (1 M) till pH = 12. The resulting powder was washed using deionized water, dried, and then calcinated at 900 °C for 3 h. Finally, the resulting powder was gathered and kept in a desiccator until characterization and electrocatalysis work.

### Preparation of bare and modified carbon paste electrodes

The bare CPE (BCPE) was prepared as described previously elsewhere [12, 13]. Meanwhile, Ca_2_CuO_3_ NS/CPE was made by combining pure graphite, paraffin wax, and Ca_2_CuO_3_ NS by the percentage 60:25:15, respectively. The resulting mixture was heated to create a homogenous paste, and then the latter was inserted in a cylindrical plastic tube with an internal diameter of 3.91 mm. A copper wire was inserted and fixed in the paste to establish electrical contact with the external circuit. To activate the manufactured electrodes, a repetitive cyclic voltammetry between 0.0 and 1.0 V in a BR buffer solution (pH = 3.2) was applied till a fixed voltammogram was attained.

### Preparation of real samples

#### Pharmaceutical samples

Ofloxacin® (400 mg) and Ciprofloxacin® (500 mg) were used as pharmaceutical samples for OFL and CIP, respectively. Five tablets of both samples were weighed and powdered in a mortar individually. An appropriate amount of the resulting powder was dissolved in deionized water and filtered to remove inactive excipients. To attain the necessary drug concentration, the resultant solution was properly diluted with deionized water.

#### Human serum samples

Blood sample obtained from healthy volunteer was supplied by South valley University Hospital (The code of ethics of South Valley University was applied). To remove protein residues, 2.0 mL of methanol was added to 1.0 mL of the real sample that, was then diluted using PBS (0.1 M, pH 4.0) and subsequently centrifuged at 5000 rpm for 10 min. The supernatant was then saved for analysis. The quantification analysis was carried out employing the standard addition method.

## Results and discussion

### Characterization of Ca_2_CuO_3_ NS

**Fig. 1:**
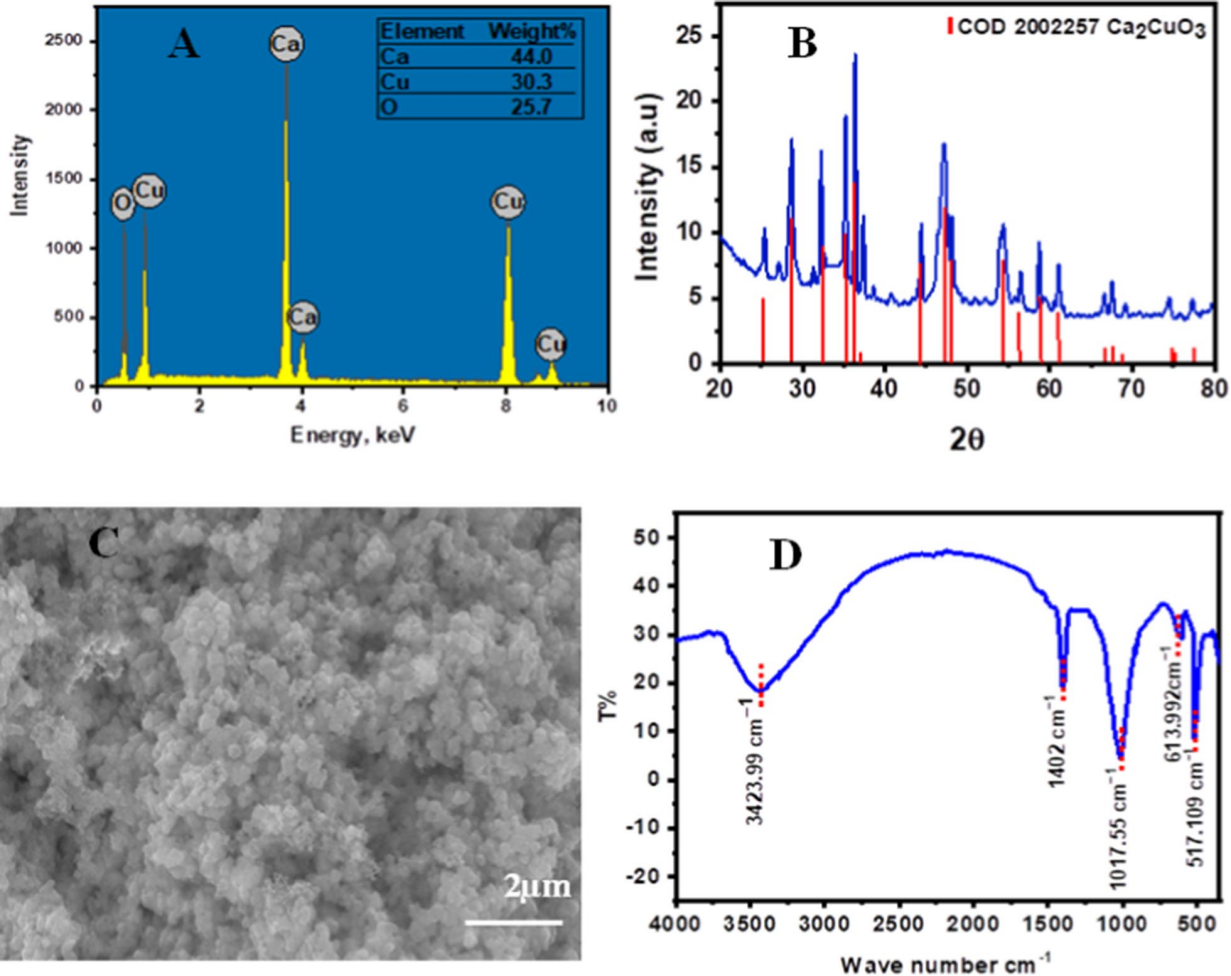
EDX pattern of Ca_2_CuO_3_ (**A**), XRD patterns of Ca_2_CuO_3_ (**B**), SEM image of Ca_2_CuO_3_ (**C**), and FTIR spectrum of Ca_2_CuO_3_ (**D**)

To determine the chemical composition and purity of the synthesized Ca_2_CuO_3_ composite, EDX analytical measurement was used. Figure 1A shows the EDX pattern of Ca_2_CuO_3_; it is evident that the only components present are Ca, Cu, and O, indicating the high purity of the synthesized composite. The crystalline structure of the generated Ca_2_CuO_3_ NS was examined using XRD pattern analysis, shown in Fig. 1B. According to the standard COD (2002257 Ca_2_CuO3), the obtained diffraction peaks were clearly attributed to the orthorhombic phase of Ca_2_CuO_3_ [20]. Using the Debye–Scherrer equation [26], the average crystalline size of the produced nanocomposite was calculated and found to be 42.3 nm. SEM was applied to characterize the morphological and visual characteristics of Ca_2_CuO_3_. Figure 1C demonstrates the morphology of Ca_2_CuO_3,_ confirming the prepared composite’s nanostructure and coral reef’s structure. In order to examine the chemical structure of the produced spinal, FT-IR spectroscopy was utilized as well. As shown in Fig. 1D, the vibrations of Cu–O, Ca–O, and Ca–O–Cu have peaks at 517.109, 613.922, and 1017.55 cm^−1^, respectively. The stretching vibration of unidentate carbonate caused by the adsorption of atmospheric CO_2_ is shown by the absorption peak located at approximately 1402 cm^−1^ [27]. Additionally, a broad adsorption band located at about 3423.99 cm^−1^ is related to –OH vibration due to the absorbed water molecules when the prepared sample comes into contact with the atmospheric air [22, 27].

### Electroactive surface area measurements

**Fig. 2:**
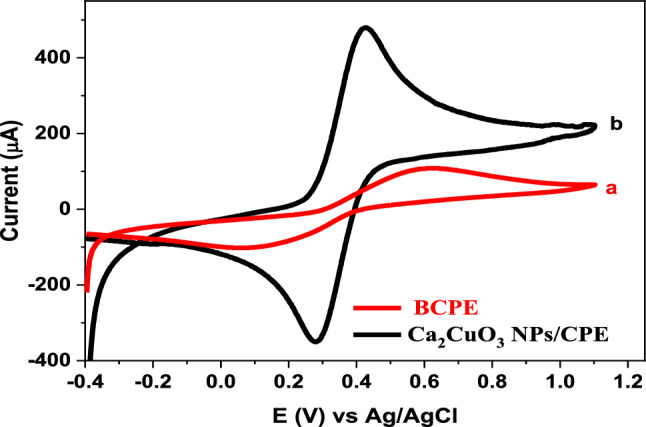
Cyclic voltammograms of 1.0 mM [Fe(CN)_6_]^3−/4−^ in 0.1 M KCl and a scan rate of 50 mV/s at BCPE (**a**), and Ca_2_CuO_3_ NS/CPE (**b**)

Cyclic voltammograms at BCPE and Ca_2_CuO_3_ NS/CPE in KCl (0.1 M) supporting electrolyte containing 1.0 mM of [Fe(CN)_6_]^3−/4−^ at a scan rate of 50 mV/s are shown in (Fig. 2). At both electrodes, [Fe(CN)_6_]^3−/4−^ displayed a reversible redox reaction with separation peak potentials (Ep) for BCPE and Ca_2_CuO_3_ NS/CPE of 0.512 V and 0.146 V, respectively. Additionally, compared to BCPE, the current signal at Ca_2_CuO_3_ NS/CPE is raised by 4.32 times. This finding demonstrates how Ca_2_CuO_3_ NS enhanced the electrochemical signal at the modified electrode and decreased the charge-transfer resistance, which promoted the electrochemical response of CPE. Thus, according to the obtained result, Ca_2_CuO_3_ NS/CPE has good electrocatalytic activity and can be applied for the appropriate analytical applications. The active surface area (A) for both applied electrodes was calculated using the Randles–Sevcik formula (Ip = (26.9 × 10^4^) n^1.5^ AD_R_^0.5^ υ^0.5^ Co) [14] and found to be 0.01 cm^2^, and 0.067 cm^2^ for BCPE and Ca_2_CuO_3_ NS/CPE, respectively. These results indicate that the Ca_2_CuO_3_ NS/CPE has a significantly higher electroactive surface area than BCPE.

### Electrochemical behaviors of CIP and OFL at BCPE and Ca_2_CuO_3_ NS/CPE

**Fig. 3:**
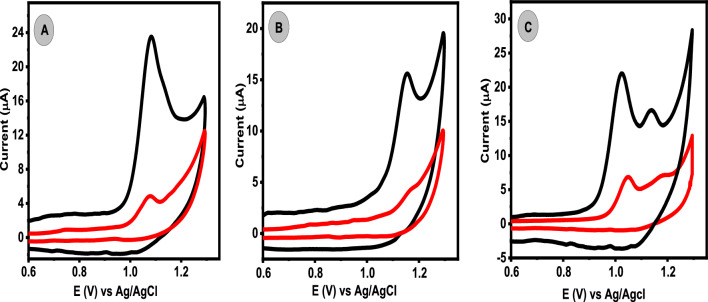
Cyclic voltammograms of 1.0 µM OFL (**A**), 0.5 µM CIP (**B**), and the mixture of the two drugs (**C**) in the presence of (0.1 M, pH = 4) PBS scan rate of 50 mV/s. Redline for BCPE and black line for Ca_2_CuO_3_ NS/CPE

As shown in Fig. 3, the electrochemical behaviors of OFL and CIP at BCPE and Ca_2_CuO_3_ NS/CPE were studied using the CV method in PBS (pH 4.0). The individual cyclic voltammograms for (1 µM) OFL and (0.5 µM) CIP at both electrodes were shown in Fig. 3A, B, as shown, both analytes exhibited an irreversible oxidation peak. Furthermore, it is obvious that the addition of Ca_2_CuO_3_ NS to CPE significantly increased the peak current signals, almost 4.7 times for OFL and 3.8 times for CIP compared to the anodic current signal at the surface of BCPE for the same concentrations. This enhancement may be attributable to the better adsorption capacity and strong catalytic activity of Ca_2_CuO_3_ NS, both are expected to improve the accumulation of the target analyte molecules at the modified electrode surface and expose more CPE active surface area [20, 28]. Obviously, the mixture of OFL and CIP drugs displayed two well-defined and sensitive anodic peaks with enhanced current response at the Ca_2_CuO_3_ NS/CPE, as shown in Fig. 3C. Additionally, the peak separation (ΔEp) value for both analytes was found to be 130 mV which is enough peak to peak separation that permitted the simultaneous determination of OFL and CIP at the Ca_2_CuO_3_ NS/CPE.

### Effect of the pH

**Fig. 4:**
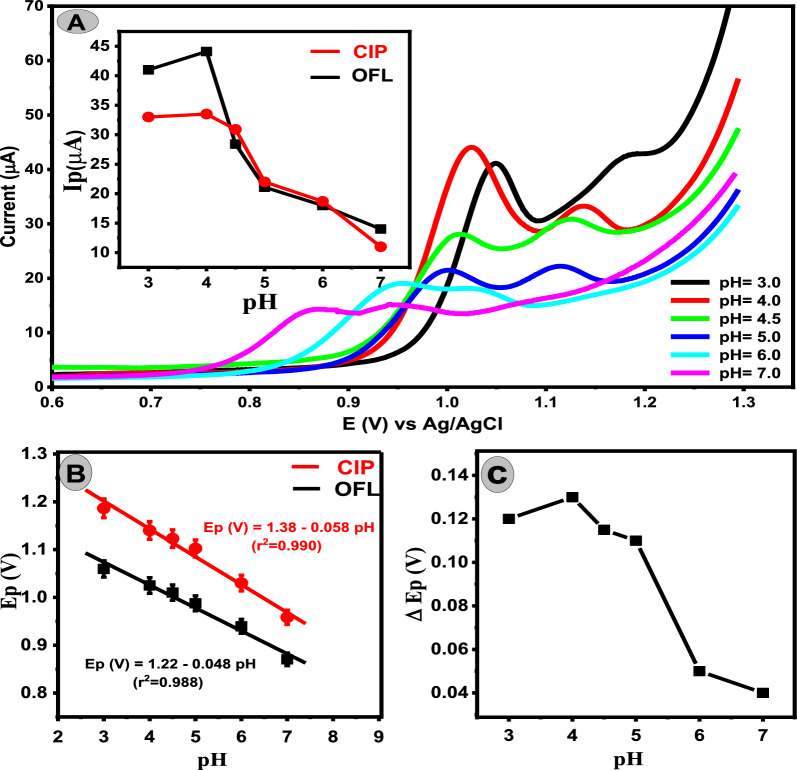
**A** Linear sweep voltammograms of 5.0 μM OFL and 3.0 μM CIP mixture in PBS at different pH values and a scan rate of 50 mV/s on Ca_2_CuO_3_ NS/CPE. Insite relation between peak current of OFL (Black line) and CIP (Red line). **B** Dependence of Ep on pH for both drugs. **C** Dependence of potential peak separation ΔEp on pH

Linear sweep voltammetry (LSV) was utilized to investigate the impact of PBS pH value on the anodic response of 5 μM OFL and 3 μM CIP at the Ca_2_CuO_3_ NS/CPE surface Fig. 4. It was observed that the highest anodic current signals of OFL and CIP with maximum ΔEp resulted at pH 4. Thus, PBS of pH = 4 was used as the supporting electrolyte for further experiments. Additionally, the observation showed that by increasing the pH value from pH = 3 To pH = 7, the Ep shifted towards more negative values, illustrating the participation of protons in the oxidation process [29]. The linear relationship between Ep and pH for OFL and CIP are Ep(V) = 1.22–0.048 pH (r^2^ = 0.990) and Ep(V) = 1.38–0.058 pH (r^2^ = 0.988), respectively. The slopes for OFL and CIP, 0.048 and 0.058, respectively, show that the equivalent protons and electrons were involved in the electrochemical reaction [30]. These investigations follow the electrochemical reaction mechanisms of OFL and CIP as described in Scheme 1 [31, 32].

### Effect of the scan rate

**Fig. 5:**
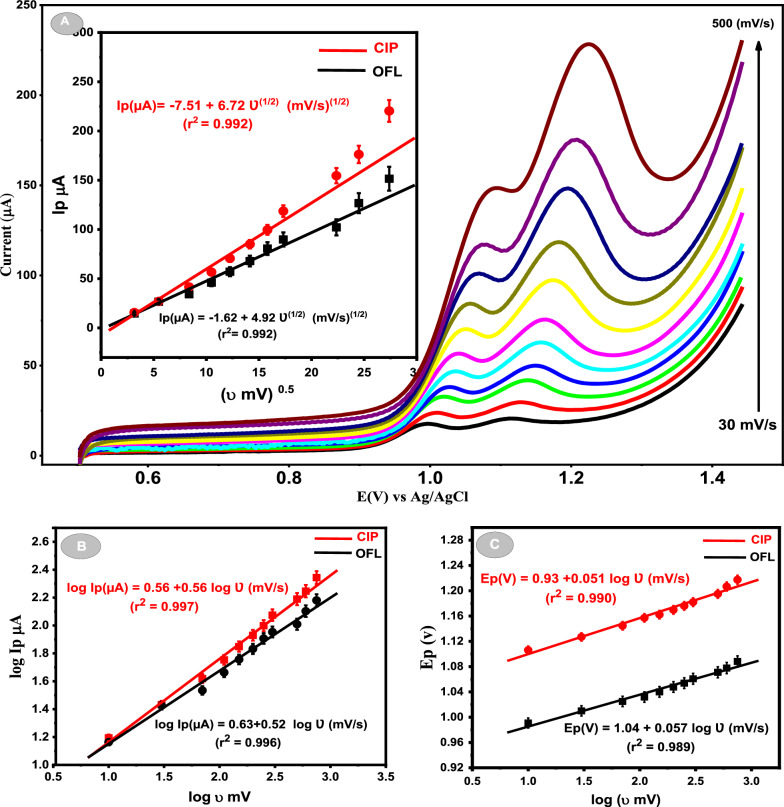
**A** LSVs of 5 μM OFL and 3 μM CIP mixture in PBS (pH = 4) at various scan rates at Ca_2_CuO_3_ NS/CPE. Insite dependence of Ip (µA) on Ʋ^0.5^ (mV/s)^0.5^ for both OFL (Black line) and CIP (Red line). **B** Relation between log Ip (µA) and log Ʋ (mV/s) for both drugs. **C** Relation between Ep (V) and log Ʋ (mV/s)

The effect of scan rate on the peak response. of 5.0 μM OFL and 5.0 μM CIP was investigated in PBS. Different scan rates were applied ranging from 30 to 500 mV in the presence of PBS and Ca_2_CuO_3_ NS/CPE as a working electrode. The recorded linear sweep voltmmograms are shown in Fig. 5. In which one can observe that on increasing the scan rate up to 500 mV/s, Ip of both drugs gradually increased, and Ep shifted positively. The results showed that Ip for both OFL and CIP is proportional to the square root of scan rate (Ʋ^1/2^). Figure 3 inset demonstrates that the electrode reactions of OFL and CIP are diffusion controlled.

Meanwhile, on plotting log Ip versus log Ʋ (Fig. 5B), a straight line with calculated slopes of 0.52 and 0.59 for OFL and CIP, respectively, the resulting slopes are close to 0.5, which indicates that the electrochemical oxidation of OFL and CIP at the Ca_2_CuO_3_ NS/CPE is diffusion-controlled [33]. Furthermore, Ep also showed a good linear relationship against the log Ʋ for the two drugs (Fig. 5C).

According to Laviron’s theory, as cited in [14], Ep/log(Ʋ) can be described for an irreversible electrode process by the following equation at 25 °C.

1 $$\frac{{\varvec{\Delta Ep}}}{{\varvec{log\upsilon } }} = \frac{{{\mathbf{0.059}}}}{{\varvec{\alpha n}}},$$

where n represents the number of electron transfers in the rate-determining step and α symbolizes the charge transfer coefficient. According to Eq. (1), the αn was calculated to be 1.03 and 1.15 for OFL and CIP, respectively. Generally, α is granted to be 0.5, and n was found to be 2.06 for OFL and 2.3 for CIP, so the number of transfer electrons during the electrochemical oxidation for both analytes can be considered to equal 2. Due to the similarities in structure between OFL and CIP reported in the literature [31, 34], the probable oxidation reactions of OFL and CIP at Ca_2_CuO_3_ NS/CPE surface can describe as follows:

For OFL
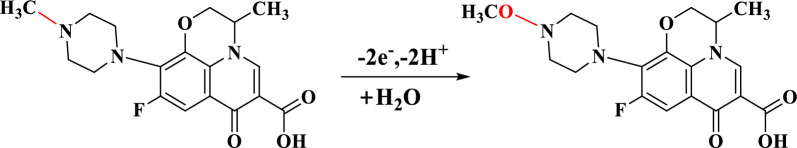


For CIP
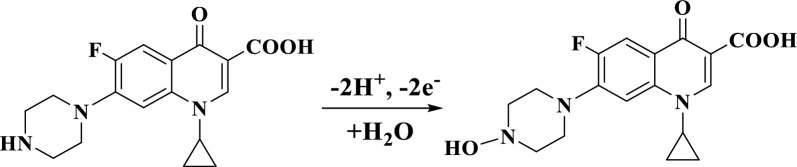


### Chronoamperometric study

**Fig. 6:**
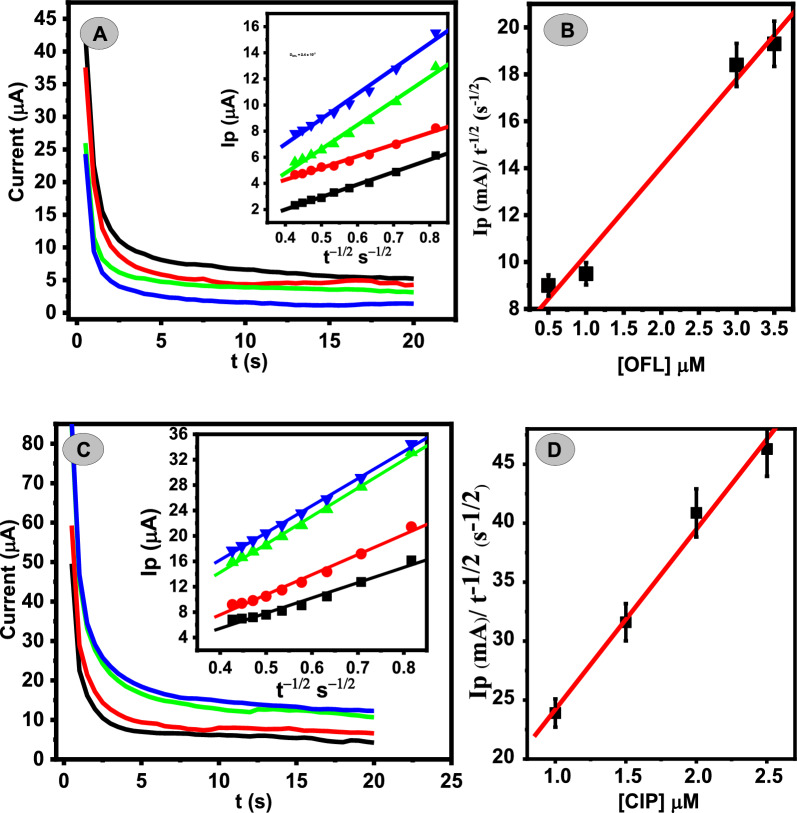
**A** Chronoamperograms obtained at Ca_2_CuO_3_ NS/CPE in the presence of different OFL in PBS (0.1 M, pH = 4), inset dependence of OFL peak currents on the t^−1/2^ derived from the chronoamperogram data, **B** plot of the corresponding slopes against OFL concentrations, **C** chronoamperograms obtained at Ca_2_CuO_3_ NS/CPE in the presence of different CIP concentrations in PBS (0.1 M, pH = 4), inset dependence of CIP peak currents on the t^−1/2^ derived from the chronoamperogram data, **D** plot of the corresponding slopes against CIP concentrations

The chronoamperometric method was used to calculate the diffusion coefficient (D) values for the electrochemical oxidation of OFL and CIP at Ca_2_CuO_3_ NS/CPE surface by applying the Cottrell equation [35] Eq. (2)

2 $${\varvec{Ip}} = {\varvec{nFAD}}^{{{\mathbf{1}}/{\mathbf{2}}}} \varvec{C\pi }^{{ - {\mathbf{1}}/{\mathbf{2}}}} {\varvec{t}}^{{ - {\mathbf{1}}/{\mathbf{2}}}} ,$$

where A is the geometric surface area of the fabricated electrode (0.12 cm^2^), C symbolizes the analyte concentration (mM), and t represents the time elapsed (s). (Fig. 6) demonstrates chronoamperograms of various concentrations of OFL (0.5–3.5 µM) and CIP (1.0–2.5 µM) at the potentials of 1.01 V and 1.14 V for OFL and CIP, respectively, in PBS (pH = 4.0). The relation between I and t^−1/2^ resulted in straight lines for various concentrations of both analytes. The diffusion coefficient was found to be 2.40 × 10^–7^ cm^2^/s and 4.03 × 10^–6^ for OFL and CIP, respectively.

### Individual voltammetric determination of OFL and CIP

**Fig. 7:**
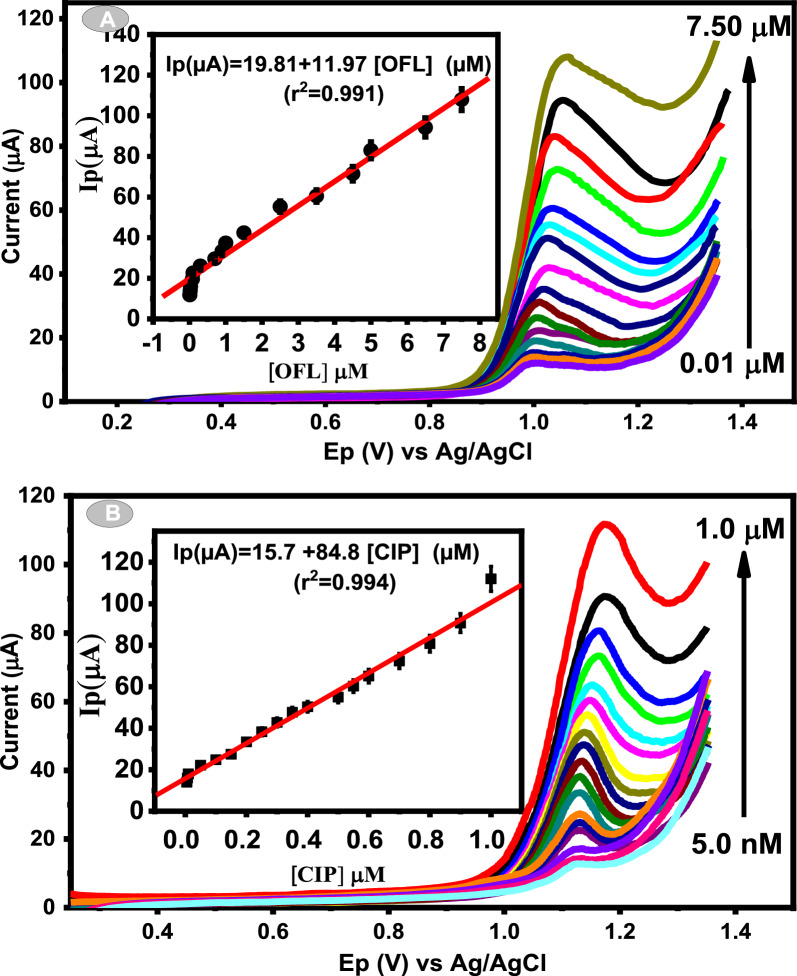
**A**, **B** DPVs of individual detection of OFL (**A**) and CIP (**B**) at Ca_2_CuO_3_ NS/CPE in PBS (pH = 4). The inset in **A** and **B**: the corresponding calibration curve of OFL and CIP with different concentrations, respectively

DPV was utilized to perform the individual electrochemical responses of OFL and CIP at Ca_2_CuO_3_ NS/CPE (the DPV parameters were optimized at a pulse height of 40 mV, pulse width of 0.005 s, step height of 15 mV, and step width of 0.01 s). As illustrated in Fig. 7, the anodic current signals increased linearly by increasing the concentration in the 0.01–7.5 μM range and 0.005–1.0 μM for OFL and CIP, respectively. LOD values were calculated to be 0.028 μM and 0.014 μM for OFL and CIP, respectively, according to S/N = 3. Additionally, LOQ values were estimated to be 0.094 μM and 0.046 μM, respectively according to S/N = 10. The outcomes demonstrated the significant sensitivity of the modified sensor toward the electrochemical oxidation of both drugs.

The proposed method is compared to previous methods used to determine OFL and CIP in Table 1. As illustrated in Table 1, different modifiers have been used to enhance the voltammetric performance of various sensors toward OFL and CIP detection. However, most of them are characterized by their relatively high cost. On the other hand, we developed an ecofriendly and cheap modified sensor in our work. Moreover, the developed method in this study showed lower LOD for OFL and CIP than most reported studies. As a result, due to the incontestable merits of the electrochemical method, Ca_2_CuO_3_ NS/CPE will be a feasible voltammetric sensor for the quantification of OFL and CIP.

**Table 1:** Comparison of the linearity range and LOD for detecting OFL and CIP using various electrodes

Method	Working electrode	Analyte	Linear range [µM]	LOD [µM]	References
DPV	P-L CuO:Tb^3+^ NS/GCE	OFL	0.01–800.0	0.0019	[36]
DPV	TiN-gC/GCE	OFL	0.05–1.0	0.016	[37]
DPV	LGCE	OFL	25–200	0.75	[38]
DPV	Cu_2_O/NG/Nafion/GCE	OFL	1.0–55	0.60	[39]
DPV	nAu@Ti_3_C_2_Tx/PABSA/GCE	OFL	0.05–500	0.037	[32]
DPV	Ca_2_CuO_3_ NS/CPE	OFL	0.01–7.5	0.028	Present Work
				0.027^a^	
DPV	Ch-AuMIP/GCE	CIP	1–100	0.21	[40]
DPV	MIP/rGO/GCE	CIP	10 -104	1.7	[41]
DPV	AuNPs/AC/GCE	CIP	0.005–0.025	0.002	[42]
SWAdAS	NiONPs-GO-CTS: EPH/GCE	CIP	0.040–0.97	0.006	[43]
DPV	Ca_2_CuO_3_ NS/CPE	CIP	0.005–1.0	0.014	Present work
				0.012^a^	

P-L CuO: Tb^3+^ NS, GCE, TiN-gC, LGCE, NG, PABSA, Ch-AuMIP, MIP/rGO, AuNPs/AC, and GO-CTS: EPH, mean peony-like dual-functional terbium doped copper oxide nanostructure, Glassy carbon electrode, Titanium nitrides nanoparticles/graphitic carbon, Laser modified Glassy carbon electrode, Poly-p-aminobenzene sulfonic acid, chitosan- gold molecular imprinted polymer, molecular imprinted polymer/reduced graphene oxide, Gold nanoparticles/Activated carbon, and graphene oxide-chitosan polysaccharide: EPH crosslinked agent, respectively

^a^Simultaneous detection

### Simultaneous determination of OFL and CIP at Ca_2_CuO_3_ NS/CPE

**Fig. 8:**
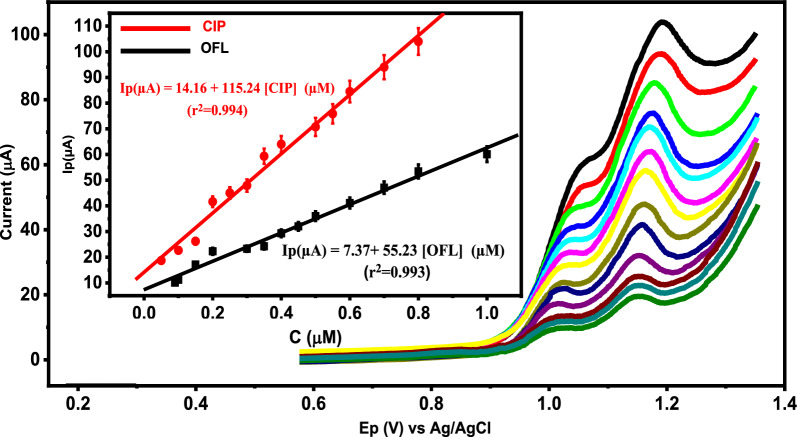
DPVs of Ca_2_CuO_3_ NS/CPE at different concentrations of OFL and CIP. Insets the calibration curves for the simultaneous determination of OFL (black line) and CIP (red line)

To examine the applicability of Ca_2_CuO_3_ NS/CPE for the simultaneous detection of OFL and CIP in a mixture, DPV has been chosen to study the electrochemical oxidation responses of both drugs by the simultaneous change in the concentrations of OFL and CIP. As indicated in Fig. 8, Ip for both analytes increases linearly by increasing their concentrations in the range of 0.09–1 μM for OFL and 0.05–0.8 μM for CIP. LOD was calculated to be 0.027 μM and 0.012 μM for OFL and CIP, respectively, according to S/N = 3. These findings illustrate that Ca_2_CuO_3_ NS/CPE can be successfully applied for the simultaneous detection of OFL and CIP.

### Interference, repeatability, and stability studies

The influences of some potential interference species of inorganic ions and organic compounds were studied under optimal conditions. The obtained results verified that about 150 folds of magnesium stearate, starch, lactose, poly (ethylene glycol), TiO_2_, cellulose, talc, Zn^2+^, Ca^2+^, Na^+^, K^+^, Fe^3+^, Cl^−^, SO_4_^2−^, and about 100 times excess of ascorbic acid, uric acid, dopamine did not influence the electrochemical response of both drugs when the tolerance limit was considered as an ± 5% error. Thus, it can be confirmed that Ca_2_CuO_3_ NS/CPE showed strong selectivity with no obvious interference effect on the detection of both fluoroquinolones.

The repeatability of the applied electrode was evaluated by measuring the same concentration using the same electrode for 6 successive measurements, and the calculated relative standard deviation (RSD) was found to be 3.9% for OFL and 2.43% for CIP, which indicated that the repeatability of Ca_2_CuO_3_ NS/CPE is satisfied.

The storage stability of Ca_2_CuO_3_ NS/CPE was also examined by the DPV response. The sensor was rinsed with PBS after each measurement and then stored at room temperature (~ 25 °C). A lowered voltametric response of the Ca_2_CuO_3_ NS/CPE of 3.9% to 5.1% was noted after 21 days for CIP and OFL, respectively, which demonstrates the high stability of Ca_2_CuO_3_ NS/CPE.

### Analytical applications

To confirm the applicability of the fabricated electrode in clinical applications, the Ca_2_CuO_3_ NS/CPE was utilized for detecting OFL and CIP concentrations in the pharmaceutical formulations Ofloxacin® (400 mg) and Ciprofloxacin® (500 mg), respectively. Additionally, Ca_2_CuO_3_ NS/CPE was utilized to detect OFL and CIP in human serum samples. As shown in Tables 2 and 3, recoveries between 97.32 and 100.40% were obtained, demonstrating a satisfactory level of accuracy for this method that may satisfy the needs of chemical analysis. Thus, the developed electrochemical sensor is applicable to determine OFL and CIP simultaneously.

**Table 2:** DPV analysis of OFL and CIP in pharmaceutical samples at Ca_2_CuO_3_ NS/CPE

Tablet brand	Added (mg/20 mL)	Founded^a^ (mg/20 mL)	RSD	^b^Recovery%
Ofloxacin® (500 mg)	20	19.65	3.78	98.25
	40	39.79	2.42	99.48
	60	58.39	1.25	97.32
Ciprofloxacin® (500 mg)	20	19.76	2.54	98.80
	40	40.06	1.79	100.15
	60	59.66	2.82	99.43

^a^ Repeated at least three times

^b^ Recovery = FoundedAdded × 100

**Table 3:** DPV analysis of OFL and CIP and human serum samples at Ca_2_CuO_3_ NS/CPE

Analyte	Added µM	Founded^a^ µM	RSD	^b^Recovery (%)
OFL	0	0	–	–
	10	9.85	2.59	98.50
	20	19.59	2.41	97.95
	30	30.12	1.98	100.40
CIP	0	0	–	^–^
	5	4.95	3.36	99.00
	10	10.02	3.22	100.23
	15	14.75	2.18	98.33

^a^Repeated at least three times

^b^$$Recovery = \frac{Founded}{{Added }} \times 100$$

## Conclusions

This work achieved the conversion of eggshell waste into useful Ca_2_CuO_3_ NS with high catalytic activity. For the first time, the prepared Ca_2_CuO_3_ NS was utilized to promote the electrocatalytic activity of CPE toward the simultaneous detection of two different fluoroquinolones antibiotics, OFL and CIP. The manufactured sensor was made with a special composition that is stable, repeatable, cheap, simple, sensitive, selective, and accurate towards both medicines. The individual detection of OFL and CIP can be performed in the range of 0.01–7.5 μM for OFL and 0.005–1.0 μM for CIP. LOD values were calculated as 0.028 μM and 0.014 μM for OFL and CIP, respectively. Additionally, the simultaneous detection of the two antibiotics was performed in the linear range of 0.09–1 μM for OFL and 0.05–0.8 μM for CIP with the LOD of 0.027 μM and 0.012 μM, respectively. Finally, Ca_2_CuO_3_ NS/CPE, with the aid of DPV offered an effective and inexpensive sensor for the successful simultaneous detection of OFL and CIP in pharmaceutical and human serum samples with an acceptable recovery value of (97.32% to 100.40%).

## Data Availability

Data are available in request.

## Abbreviations

Ca_2_CuO_3_ NS: Ca_2_CuO_3_ nanostructure
CPE: Carbon paste electrode
OFL: Ofloxacin
CIP: Ciprofloxacin
FQs: Fluoroquinolones
PBS: Phosphate buffer solution
CV: Cyclic voltammetry
LOD: Limit of detection
LOQ: Limit of quantification
LSV: Linear sweep voltammetry
DPV: Differential pulse voltammetry
